# Supplementary material for: Are carbon emissions trading and green financial instruments synergistic? -Comprehensive quantitative research based on content analysis
Source: PLoS One. 2024 Mar 7;19(3):e0298601. doi: 10.1371/journal.pone.0298601 (PMC10919601; doi:10.1371/journal.pone.0298601)
Supplement: S1 Data — (ZIP) [file pone.0298601.s001.zip › Data/地级市Z_F工作报告.zip!/║╙─╧/╓ú╓▌╩╨/└┤╫╘╣1⁄2╓┌║┼í╛┬φ┐╦╩2╛▌═°í┐_2006.docx]

各位代表：

　　现在，我代表市人民政府，向大会作工作报告，请予审议，并请各位政协委员和列席人员提出意见。

　　2005年和“十五”政府工作回顾

　　2005年，市人民政府在省委、省政府和市委的正确领导下，带领全市人民，以科学发展观统领经济社会发展全局，以开展保持共产党员先进性教育活动为动力，按照“加快发展，保持稳定，为民谋利”的基本思路和要求，开拓创新，真抓实干，全市经济社会实现了又快又好的发展，圆满完成了市十二届人大二次会议确定的各项目标任务。

　　初步统计，全市实现生产总值1650亿元，增长15.8%，人均生产总值达到2850美元；地方财政收入151亿元，增长30.7%；全社会固定资产投资820亿元，增长28.6%；社会消费品零售总额706.7亿元，增长14.5%；非公有制经济完成增加值875亿元，增长22%，占全市生产总值的比重达到53%。城镇居民人均可支配收入达到10640元，增长13.6%；农民人均纯收入达到4774元，增长14.1%。

　　工业经济快速增长，产业竞争力不断增强。全部工业增加值完成759.7亿元，增长20.8%，其中规模以上工业增加值569.7亿元，增长24.2%。销售收入超亿元企业达到306户，比2004年增加110户。“工业项目建设年”活动成效显著，工业投入创历史新高，项目投资完成300.9亿元，增长43.3%，中铝河南分公司70万吨氧化铝等78个重点工业项目竣工投产。铝工业、装备制造、汽车、食品加工等优势产业竞争力不断增强。氧化铝产量突破170万吨，位居亚洲首位；宇通公司豪华客车、城市公交客车产销量居全国第一，郑州日产的高档皮卡国内市场占有率达40%；速冻食品产量达到32.3万吨，占全国总产量的50%以上。“宇通”、“三全”、“思念”成为“中国500最具价值品牌”。

　　商业新型业态发展较快，现代服务业迅速成长。传统商贸流通业加快升级，连锁业得到快速发展，麦德龙、五星电器等知名商业零售企业先后落户我市，家世界、正大易初在我市的扩张步伐不断加快，圃田建材等交易园区建设进展顺利。现代物流业发展迅速，国际航空货运中心工程正在抓紧建设，公路港、铁路港、航空港“三位一体”的物流体系逐步形成。总投资22亿元的郑州国际会展中心建成并投入使用，已成功举办了第十一届郑交会、中原国际工业博览会等大型展会。我市入选中国会展业最佳会展城市、中国最具潜力的会展新锐城市，郑州国际会展中心被授予中国展馆新锐奖。文化产业快速发展。积极实施文化精品名牌带动战略，打造了一批艺术精品，文化产品的市场竞争力全面提升。大型原创舞剧《风中少林》荣获全国舞蹈界最高奖“荷花奖”金奖，入选国家舞台艺术精品工程，已成功赴台湾演出，并签订赴美国巡演协议。旅游业继续保持良好的发展态势，全年旅游总收入达到178亿元，同比增长28.4%。郑交所白糖期货成功上市，保险、中介等服务业迅速发展。

　　农村经济持续增长，现代农业发展步伐加快。认真贯彻中央1号文件精神，狠抓农业结构调整，农业现代化、产业化水进一步提高，农村经济全面发展。全市农林牧渔业增加值72亿元，增长7%。现代畜牧业发展迅速，畜牧业增加值28.4亿元，占农林牧渔业增加值的39.5%。农业龙头企业不断发展壮大，年销售收入总额突破110亿元。新建奶牛养殖小区20个，新增种植业无公害生产基地15万亩，建成12个全省领先的现代农业示范园区。切实加大对农业的投入，全年安排财政支农资金5.7亿元，增长22.1%。全面落实免征农业税政策和农村税费改革有关配套措施，免征农业税9238万元，兑付粮食直补、良种补贴、农机具购置补贴5357万元。农村道路、水利等基础设施建设进一步加强，新建、改建农村公路1963公里，解决了6万人的饮水安全问题。“三个一万”工程全面完成，建设沼气池2.4万个，西部缺水山区新建集雨节灌水窖1万个，巩固和解决1.2万贫困人口脱贫。森林生态城工程建设步伐加快，全市新增造林面积25.6万亩。

　　城市建设力度加大，城市面貌明显改观。中心城区保护改造“三年一大变”目标基本完成。续建、新修南三环东段、新柳路等道路14条(段)，西三环?D化工路立交已建成通车，北三环?D文化路立交等工程进展顺利，改造支路背街小巷121条，对全市红线宽20米以上的道路全部进行了整治。新开、优化公交线路55条，新购运营车辆303台，新增供水管道160公里，新建、改造雨污水管网49.7公里，市区新建公厕102座。全年新增绿地700余万平方米，新建公园8个、游园42个。五龙口污水处理厂一期日处理10万吨工程建成投入运行，马头岗污水处理厂全面开工建设，垃圾综合处理场主要填埋场建成投入使用。绕城高速公路已建成通车。“四城联创”成效明显，创建国家园林城市工作胜利完成，创建国家卫生城市工作已通过技术考核，创建国家环境保护模范城市工作大多数考核指标已达标。

　　郑东新区建设“三年出形象”目标基本实现。累计完成投资232亿元，引进项目170个，开工项目120个。基础设施建设明显加快，续建和新建道路137条(段)，其中96条(段)基本具备通车条件，通车里程达到102公里。新区热源站开始供暖，中心湖喷泉景观及水幕电影投入使用。中央商务区形象基本显现，入驻项目59个，其中52个开工建设，内、外环正在建设的47栋高层中31栋已结顶；河南艺术中心的美术馆、艺术馆主体已封顶，会展宾馆项目已与上海绿地集团签约。龙湖南区、商住物流区、龙子湖区、科技园区建设取得新进展。

　　中小城市和小城镇建设取得新成就。对中小城市和小城镇建设的投入力度不断加大，全年500万小城镇建设专项资金全部奖补到位，重点支持31个市级重点镇建设。

　　各项改革稳步推进，对外开放水平逐步提高。国有企业改革步伐加快，全年完成176户企业改制任务。中原制药厂重组进入实质性操作阶段，一、四棉公司改制程序启动，三棉改制后新公司正式运营，电缆、白鸽、友谊等企业集团改制进展顺利。永通特钢在香港成功上市。以郑州铁路系统、郑煤集团等企业的32所中小学交接为重点的企业主辅分离工作已经完成。粮食流通体制改革稳步进行，全市粮食流通企业职工全部实现身份转换。煤炭资源整合成效显著，关闭了240多个小煤矿。乡镇机构改革基本完成，撤并乡镇15个，分流行政事业人员3535名。投融资体制改革进展顺利，多元化投融资格局初步形成。农村信用社改革稳步推进，支农实力明显增强。文化体制改革迈出新步伐，对新组建的郑州歌舞剧院进行了股份制改造，《禅宗少林?音乐大典》和大型舞剧《洛神》实行市场化运作。

　　对外开放进一步扩大。注重优化经济发展环境，健全完善政策体系和招商引资激励机制，有效调动了全市上下招商引资的积极性。浙江绿城等一批招商大项目落户郑州，引进市外境内资金达102亿元。全年新增外商投资企业118家；实际利用外资3.4亿美元，增长38.6%；进出口总额达到11亿美元，增长41.2%。组织参加豫港投资贸易洽谈会、中国河南(旧金山)经贸文化周等经贸活动，招商活动取得明显成效。积极实施“走出去”战略，一批企业到国外投资，有13家企业进驻非洲塞拉里昂工贸园区，海外市场进一步拓展。出口加工区封关运行良好，郑东新区、高新技术产业开发区、经济技术开发区招商工作取得新进展，加州工业城项目正在加紧推进。区域经济交流与合作明显加强。

　　各项社会事业全面进步，就业和社会保障工作取得新进展。继续实施科教兴郑战略，科技创新体系建设得到加强，科技进步对经济增长的贡献率达到50%。学校布局和结构调整加快，教育资源配置进一步优化。首次对县(市)区教育工作进行督导评估。22所新建中小学全部竣工，入学困难得到缓解。成功举办了全国第九届中学生运动会。电影《任长霞》在全国放映，《任长霞》电视剧和戏曲相继播出。群众文化活动广泛开展，广场文化、社区文化等呈现出良好的发展态势。疾病预防控制和医疗救治体系建设明显加强，建成郑州市紧急医疗救援中心，形成覆盖城乡的救援体系。建成7所示范乡镇卫生院，新型农村合作医疗试点工作进展顺利。人口和计划生育工作得到加强，全市人口出生率低于省下达控制目标，流动人口计生工作经验在全国推广。经济普查取得阶段性成果。社区建设工作力度不断加大。第五届村委会换届选举工作圆满完成。民兵预备役建设和优抚安置工作进一步加强。

　　就业再就业工作成效明显，实现城镇就业再就业12.5万人，农村劳动力转移就业14.1万人。全年各级财政投入社会保障资金14.3亿元。在全国率先实行市属国有破产和困难企业退休人员大病统筹医疗保险，国有企业退休职工生活补贴、中小学退休教师待遇等问题得到解决。社会保障和社会救助体系进一步完善，在全省率先实行农村居民最低生活保障制度，城乡低保做到了动态管理下的应保尽保。新开工经济适用住房91.2万平方米，竣工47.8万平方米，廉租住房覆盖率达到符合条件“双困家庭”100%。救灾救济工作扎实有效。流浪乞讨人员救助管理工作创出新模式，流浪儿童救助保护在联合国人权工作会议上得到肯定。劳动监察力度不断加大，农民工利益得到切实保护。

　　民主与法制建设得到加强，社会大局保持稳定。自觉接受人民代表大会及其常委会监督，接受人民政协民主监督，认真听取民主党派、工商联、无党派人士、各人民团体的意见。全年办复人大代表议案、建议和政协委员提案1226件。制定了政府重大事项决策规则，扩大了政务公开范围，完善了政府新闻发言人和市长接待代表、委员活动日制度，政府决策的科学性和透明度进一步提高。廉政建设和反腐败斗争取得新成效。坚持依法治市，提请通过地方性法规11件，制定规章8件。加强对规范性文件合法性审查，依法规范政府行政行为。积极宣传贯彻《信访条例》，信访秩序进一步规范。妥善化解矛盾，群体性突发事件大幅减少。不断深化“两严一创”活动，全力开展命案攻坚，大力推广技防设施建设，全面构建社会治安防控体系，全市没有发生重特大刑事案件。我市荣获“全国科技强警示范城市”。健全了安全生产监管体系，严格实行安全生产责任制。省会社会大局保持稳定，人民群众安居乐业。

　　向社会承诺为群众办好的“十件实事”，件件得到落实，群众关注的一些热点、难点问题得到切实有效解决。

　　新闻出版、广播电视、人事、统计、审计、物价、侨务、对台事务、国防教育、民族宗教、社会科学、地方志、档案管理、气象和地震监测、人防等工作都取得了新的成绩。

　　2005年全市各项目标任务的圆满完成，标志着“十五”期间我市经济社会发展跃上了一个新台阶。综合实力显著增强。“十五”期间，我市经济增长速度逐年提高，全市生产总值年均增长13.6%，增速比全省和全国分别高出2.2个和4.1个百分点，生产总值和人均生产总值都实现了翻番。地方财政收入由2000年的46亿元增加到2005年的151亿元，年均增长24.9%。在国家统计局评定的2004年度中国城市综合实力百强城市中，我市名列第23位，比上年位次前移5位。城市建设取得重大进展。城市基础设施建设投入大幅增长，5年累计完成投资309.8亿元，年均增长43.3%。建成区面积由2000年的133.2平方公里扩大到262平方公里，城镇化率由55.1%提高到59.2 %。城乡居民生活水平和质量明显提高。城镇居民人均可支配收入和农民人均纯收入年均增长分别为13.5%和10.4%。城市居民和农村居民人均住房面积分别达到23和43.7平方米，分别比2000年增加3.2和11平方米。经过“十五”的快速发展，我市在全省乃至中部地区的影响力和辐射力明显增强。

　　回顾“十五”特别是近年来的政府工作，我们深深体会到，经济社会能够实现又快又好的发展，得益于我们认真坚持和把握了以下几点：一是进一步解放思想，理清了发展思路。结合郑州发展的实际，我市提出了“拉长工业短腿、发挥商贸优势、带动现代农业”的工作思路，不断优化发展环境，加大招商引资力度，大力发展民营经济。强化了工业发展对经济增长的支撑作用，工业占全市经济总量的比重达到46%以上，比2000年提高近4个百分点。二是不断创新举措，凝聚了全市人民的力量。根据每年工作重点，制定有针对性的推进措施，狠抓了“社会各界民主评议机关和行业作风”、“两个新增160亿”、“五个一百”、 “工业项目建设年”和“四城联创”等工作，从而使全市上下方向明确、思想统一，凝聚了人心，形成了合力，聚精会神搞建设、一心一意谋发展的氛围更加浓厚。三是努力加大投入，增强了发展后劲。城市建设、工业项目、农村基础设施和各项社会事业建设的投入逐年加大，带动了全市经济快速发展。“十五”期间，我市固定资产累计投资2607亿元，一大批项目相继建成，为我市今后发展奠定了良好基础。四是继续深化各项改革，增强了发展活力。国有企业改革和主辅分离基本完成，适应市场经济的现代企业制度逐步建立，企业焕发了生机和活力。以税费改革为重点的农村综合改革推向深入，农民负担大幅减轻，种粮的积极性明显提高。强力推行行政管理体制改革，行政审批项目大幅减少，机构精简高效。五是坚持人为本、为民谋利，让人民群众更多地享受发展带来的成果。从人民群众最关心的实际问题入手，不断加大社会保障投入，“十五”期间各级财政累计投入资金49.6亿元，切实解决低收入和贫困群体的教育、就业、医疗等实际问题。人居环境明显改善，城区绿化覆盖率达到34.8%。市区空气质量二级以上天数达300天。

　　各位代表，上述成就的取得，是省委、省政府和市委正确领导的结果，是市人大、市政协支持和监督的结果，是全市广大干部群众共同奋斗的结果。在此，我代表市人民政府，向给予政府工作大力支持的人大代表和政协委员，向工作在各条战线上的工人、农民、知识分子和干部，向驻郑人民解放军、武警官兵、公安政法干警，向各民主党派、工商联、人民团体和各界人士，表示崇高的敬意！向关心和支持郑州发展的港澳台同胞、海外侨胞和国际友人，表示诚挚的感谢！

　　在看到成绩的同时，也要清醒地看到经济社会发展中一些困难和问题。一是经济结构性矛盾对经济增长的影响仍比较大，资金筹措、土地审批和环境保护等发展瓶颈问题更加突出。二是农村卫生、文化、教育等公共设施建设滞后，农民就医难、增收难等仍然是农村最迫切需要解决的问题。三是社会保障体制仍待进一步完善，城乡社会统筹、医疗保险的覆盖面有待提高。四是利用外资的规模偏小，经济外向度不高。五是就业压力仍然很大，就业岗位不能充分满足需求。六是城市管理水平有待提高，创建文明城市任务十分艰巨。七是在全省的首位度还不够高，在中原崛起乃至中部崛起中的作用有待进一步发挥。八是一些政府工作人员服务意识不强、效率不高，政府廉政建设有待继续加强。这些问题，都需要在今后的工作中切实加以改进和解决。

　　“十一五”发展的基本思路和主要目标

　　“十一五”时期是我市全面建设小康社会的关键时期。我市经济社会发展的基本思路是：坚持以邓小平理论和“三个代表”重要思想为指导，用科学发展观统领经济社会发展全局，以实现跨越式发展为目标，坚持以人为本、解放思想、深化改革、务实创新，大力实施科教兴郑、开放带动和可持续发展战略，加快工业化、城市化和农业现代化进程，优化经济结构，转变增长方式，构建先进制造业基地、现代服务业中心和现代农业示范区，建设生态郑州、平安郑州、和谐郑州，把郑州建设成为全国区域性中心城市、全国重要的现代物流商贸城市、全国重要的交通通讯枢纽城市和全国著名的历史文化名城。

　　我市经济社会发展的主要目标是：在优化结构、提高效益和降低消耗的基础上，保持较快的发展速度，生产总值年均增长13%左右；保持较快的投资增幅，全社会固定资产投资年均增长17%；经济增长质量明显提高，地方财政收入年均增长15%；资源利用效率显著提高，单位生产总值能耗比“十五”末降低20%以上，资源节约型和环境友好型社会建设取得实质性进展；人民生活质量明显改善，城镇居民人均可支配收入、农民人均纯收入年均增长8%，就业比较充分，社会保障体系比较健全；社会主义市场经济体制更加完善，社会更加和谐，民主法制建设、精神文明建设和各项社会事业全面进步。

　　面临新的形势和任务，我们必须转变发展观念，创新发展模式，提高发展质量，牢牢把握五个重点，切实把经济社会发展转入科学发展轨道。

　　一是强力推进经济结构战略性调整，加快转变经济增长方式。进一步扩大内需，调整投资和消费的关系，在扩大投资规模的同时，改善消费环境，促进消费升级，增强消费对经济增长的拉动作用。在推进工业结构优化升级的同时，加快物流、会展、文化、旅游、金融、信息等现代服务业发展，努力实现先进制造业与现代服务业的双重升级。突出集约化、规模化、集群化、生态化，促进经济增长方式从粗放型向集约型、从资源型向技术型和高端型转变，促进经济发展向三次产业协调推动转变。

　　二是注重提高自主创新能力，全面提升核心竞争力。把增强自主创新能力作为发展的战略基点和调整产业结构、转变增长方式的中心环节。不断加大投入，发展对经济增长有重大带动作用、具有自主知识产权的核心技术和关键技术，建立健全促进产学研有机结合的科技成果转化体系，社会化、专业化、网络化的科技中介服务体系，合理配置科技资源、充分激活创新要素的政策调控体系，加快制度创新、管理创新、科技创新，推动引进消化吸收再创新，建设创新型城市。

　　三是统筹城乡发展，建设社会主义新农村。高度重视“三农”工作，坚持工业反哺农业、城市支持农村，统筹城乡经济社会发展，实现合理布局、科学分工、优势互补、产业对接。坚持“多予少取放活”，从农民群众最关心、要求最迫切、最容易见效的事情抓起，加大对农业和农村投入的力度，扩大公共财政覆盖农村的范围，强化对农村的公共服务，新增教育、文化、卫生等事业经费向农村倾斜，加快建设社会主义新农村，真正使建设新农村成为惠及广大农民群众的民心工程。

　　四是继续深化改革，进一步扩大对外开放。坚持社会主义市场经济的改革方向，不断深化国有企业改革，完善现代企业制度和现代产权制度，全面推进综合配套改革，在体制机制创新方面取得实质性进展。坚持实施开放带动战略，着力发展开放型经济，进一步优化对外开放环境，健全市场体系，提高生产要素的市场化程度，整顿和规范市场秩序，营造有利于扩大开放的服务环境、法制环境、人文环境、创业环境和信用环境。充分利用国际国内两个市场、两种资源，扩大招商引资规模，提高对外开放水平，增强在扩大开放条件下促进发展的能力。

　　五是坚持以人为本，全面建设和谐社会。从解决关系群众切身利益的现实问题入手，更加注重经济社会协调发展。把扩大就业摆在经济社会发展更加突出的位置，千方百计扩大就业再就业，着力提高低收入群体的收入水平。建立健全与经济社会发展相适应的社会保障体系，全面推行新型农村合作医疗，健全覆盖城乡的社会救助体系。加快发展社会事业，加大公共服务投入。更加注重民主法制建设，正确处理改革发展稳定的关系，保持社会安定团结。

　　《郑州市国民经济和社会发展第十一个五年规划纲要(草案)》及说明，大会已印发给各位代表，请予审议。

　　2006年的主要工作

　　2006年是“十一五”规划的开局之年，是承前启后的重要一年，努力做好今年的各项工作，至关重要。今年我市经济社会发展的主要预期目标是：生产总值增长14%；地方财政收入增长15%；全社会固定资产投资增长23%；社会消费品零售总额增长14%；外贸出口增长20%，实际利用外资增长30%；城镇居民人均可支配收入、农民人均纯收入增长8%；单位生产总值能耗降低5%以上；居民消费价格总水平涨幅控制在3%；人口自然增长率控制在6‰以下；城镇登记失业率控制在4%以内，城镇新增就业12万人。为实现上述目标，应着力做好以下五个方面的工作：

　　一、推进产业结构优化升级，努力实现经济跨越式发展

　　坚持把经济结构调整和经济增长方式的转变作为关系全局的重大任务，大力发展高新技术产业，加快发展先进制造业和现代服务业，加强基础产业和基础设施建设，大力发展规模经济，实现规模效益。

　　(一)坚持扶优扶强，强力推进工业快速发展。继续开展“工业项目建设年”活动，加大工业投入，加快工业结构调整，优化产业布局，着力提高工业经济增长的整体素质和内在活力。力争规模以上工业增加值增长20%，销售收入增长25%以上。大力开展工业招商，引进一批重大战略性项目，工业投入增长30%。加快支柱产业发展，以发展高档公路客车、运动休闲车、特种专用车为重点，加快汽车零部件工业园建设，提高本地产品配套率；以推进煤电铝一体化为重点，积极开发高效连铸技术和深加工技术，着力提高终端产品收益率；以建设国内重要的食品生产加工基地为目标，努力扩大速冻食品、方便食品、高档面制品、啤酒和饮料的系列化、规模化生产；整合纺织资源，加快资产重组和改造提升，推进纺织工业快速发展。利用高新技术改造传统产业，培育具有自主知识产权的核心技术，提高传统产业的科技含量和装备水平。加快发展以晶片、税控机、保密计算机、数字视听产品等为重点的电子信息产业，大力发展光机电一体化；大力培植重点新材料工业企业和龙头企业，走新材料规模化、材料制品专业化路子。实施名牌战略，整合服装、耐材、机械制造等行业的品牌，力争再创一批国家级名牌产品和在国际上有影响的知名品牌。继续加大重点企业扶持力度，力争销售收入超百亿企业达到2家、超50亿元企业4家以上。搞好工业园区和都市工业规划，抓好郑州纺织工业基地等园区建设，推进工业集群区发展。

　　(二)发挥比较优势，大力发展现代服务业。进一步深化流通体制改革，大力发展连锁经营、物流配送等新型流通方式，培育大型流通企业。规范城市商业网点建设，积极发展社区超市、便利店。努力开拓农村市场，实施“万村千乡”工程，不断完善农村商品服务体系。着力发展现代物流业，构筑现代物流体系。加快郑州国家干线公路物流港、国际航空货运中心、铁路集装箱货运中心、铁路零担货运中心和中南邮政物流集散中心等建设，重点培育豫鑫、长通等物流企业。认真做好郑州国际会展中心运行管理工作，依托会展中心，拓展会源，扩大会展规模，提高办会办展水平。筹备举办好第十二届郑交会、全国制药装备会、全国药品交易会等一批重点展会，办好第二届世界传统武术节、2006年中国企业500强发布暨高层论坛。

　　全面落实文化产业发展政策，重点扶持大众传媒、文化旅游、文博会展、出版发行、文化娱乐等主导产业，有针对性地扶持一批文化产业龙头企业。整合文化资源，继续抓好州歌舞剧院的体制机制创新，加快豫剧院和曲剧团合并步伐。重视创意型人才培养，策划一批重大文化产业项目，突出抓好商都文化苑、黄帝文化苑建设，重点抓好《禅宗少林?音乐大典》大型实景演出工作。

　　加快旅游、房地产、金融服务业的发展。积极筹建郑州旅游服务中心，下半年投入试运营。整合旅游资源，大力发展以文化为核心的旅游业，加大A级旅游景区创建力度，努力打造旅游精品。加大旅游宣传促销力度，大力开发境内外旅游市场。积极发展城市近郊游，加快雁鸣湖景区开发。积极发展房地产业，扩大普通商品房供给。进一步深化住房制度改革，推进住房货币化分配。扩大住房公积金缴费面，简化公积金贷款程序，提高贷款比例。改善金融环境，扩大直接融资规模。积极推进市商业银行资产重组，加快区域产权交易市场建设。

　　(三)加大固定资产投入力度，积极推进重大项目建设。加强综合交通体系建设，增强中心城区与卫星城的互通能力。继续加快郑汴快速通道建设，确保年内建成通车；做好郑州至新郑快速通道建设前期工作；开工建设郑州高等级绕城公路西段和北段的改造工程，打通航海东路及中原西路；开工建设郑州至石人山等高速公路。配合做好郑州至西安高速铁路客运专线及郑州车站西出口建设工作，配合做好郑州至石家庄、郑州至武汉高速铁路客运专线和铁路客运专线枢纽站的前期工作。加快新郑机场航站楼和停机坪改造扩建，力争年内建成。配合做好南水北调中线工程规划建设。搞好能源项目建设，按照全省建设火电基地的总体规划，继续抓好新力公司2×20万千瓦热电机组扩建等项目建设，做好新密2×30万千瓦电厂等项目核准工作。加快赵家寨、王行庄、白坪矿井建设。抓好重点工业项目，开工建设安彩郑州液晶玻璃基板等一批项目，带动形成产业链。加快中铝公司铝精深加工等重大项目建设。继续落实重点项目责任制，创造良好的施工环境，加强协调和服务，确保项目顺利推进。

　　(四)完善扶持政策，加快非公有制经济发展。进一步激活民间投资，放宽市场准入，打破行业垄断，鼓励引导非公有制资本进入城市基础设施、社会事业、公用事业等领域。加强服务体系建设，为中小企业提供社会化、专业化和规范化服务，协调解决非公有制企业生产经营中存在的困难和问题。加快人才培养，积极引进各类急需人才，创新人才使用机制，用好人才，留住人才，为我市非公有制经济发展提供人才和智力支持。非公有制经济增加值增长20%，不断提高在经济总量中的比重。

　　(五)推进资源节约，大力发展循环经济。抓好煤、电、铝、建材等高耗能行业节能和企业技术改造，推广节能材料和新技术，大力调整能源消费结构。全面启动节水型社会建设试点，切实做好农村节水工程。加大耕地保护力度，继续开展“空心村”等土地整治，加大实心粘土砖淘汰力度，大力推广多层标准厂房，节约和集约利用土地。搞好对煤矿瓦斯、煤层气、矿井水等资源的回收和综合利用。尽快制订加强资源综合利用的地方性法规。编制实施循环经济发展规划，推动产业循环式组合。指导、推进各县(市)区生态工业建设和上街区国家生态工业示范园区、新郑煤炭循环经济园区的建设。

　　二、统筹城乡协调发展，加快建设社会主义新农村

　　稳定完善各项政策，从项目建设、财政支出、银行贷款等方面向农村倾斜，逐步建立以工促农、以城带乡的长效机制。按照生产发展、生活宽裕、乡风文明、村容整洁、管理民主的要求，科学规划，扎实推进社会主义新农村建设。实施“百村示范、千村整治”工程，全面推进试点工作，选派机关干部下农村帮助新农村建设，使新农村建设有一个良好开局。切实加强农村基层组织和基层民主政治建设，继续深化村务公开和民主管理。深入开展爱国卫生运动，加大村容村貌整治，不断提高农民生产生活环境质量。深入开展农村精神文明创建活动，全面提高农村文明程度。

　　强力推进现代农业建设。大力调整和优化农业产业结构，加快发展都市农业、生态农业、特色农业、创汇农业。推进现代农业示范园区建设，加快黄河滩区绿色奶业示范带和邙岭林果产业带开发。着力培育一批竞争力、带动力强的龙头企业，扶持农业产业化发展，大力发展农村新型合作组织，促进农业产业化经营。千方百计增加农民收入。积极落实各项惠农支农政策，加大良种补贴和农机具补贴力度。大力发展劳务经济，鼓励引导农民发展兼业经营。搞好农民实用技术、科技知识引导性培训。继续解决拖欠农民工工资问题。进一步做好扶贫开发工作。

　　加强农村基础设施建设。全面完成县城电网改造工程，进一步完善农村电网。多渠道筹措建设资金，确保完成“村村通”道路的改造任务。继续实施农村安全饮水工程，加大农村沼气池建设力度。加强农田水利基本建设，实行以奖代补的办法大力发展小型农田水利设施。抓好李湾、坞罗等水库除险加固工程建设，提高防汛抗旱能力。搞好村镇规划，实施一批村镇建设示范工程。

　　大力发展农村公共事业。加大农村教育投入，继续落实“两免一补”政策，增加农村中小学公用经费标准，建立农村中小学危房改造长效机制，消除D级危房，有计划地建设寄宿制学校，实施现代远程教育试点、校医室建设、农村教师素质提高等农村教育三大工程。加快农村公共卫生事业发展，加强县、乡、村三级医疗卫生服务网络和乡、村级卫生机构及队伍建设，完成全市乡镇卫生院改造任务。加强农村文化设施建设，完善公共文化服务体系，满足广大农民多层次的文化需求。

　　高度重视县域经济发展，不断落实完善支持县域经济发展的各项政策，加大资金投入力度。合理规划县域经济产业布局，扶持优势产业项目，切实加强对县(市)项目建设的指导，帮助县(市)争取项目、落实资金。引导县(市)因地制宜地发展特色经济，建设特色工业园区，扩大新郑中原食品工业园等园区的规模，加快新密曲梁服装工业园、中牟汽车零部件工业园等园区建设步伐，不断壮大工业集群区的经济总量和实力。积极推进巩义市、惠济区城乡一体化试点工作。

　　三、加强城市建设与管理，不断推进城市化进程

　　以“城市管理年”活动为载体，进一步优化人居环境，加强城市建设与管理，创建国家卫生城市和国家环保模范城市，加快城市化进程。正确处理新区建设与老城区改造发展的关系，实现城市东部与西部均衡发展。

　　(一)继续加大中心城区保护改造力度。突出古都保护和改善人居环境两大重点，做好商城遗址、文庙等文物古迹的保护修缮工作。抓好中州大道、金水东路建设，重点对花园路、未来路、中原路等10条精品街实施全方位整治，完成对省市行政区、商业中心区、商城文化区等精品街区的综合整治。进一步完善城市路网结构，抓好郑新路、秦岭路、伏牛路和北三环?D文化路立交等续建项目建设，拓宽改造郑邙公路、老郑密路等道路。对紫荆山公园、绿城广场等进行升级改造。整治城市水系，增强城市防汛能力。升级改造部分道路、桥梁等市政设施，继续抓好夜景照明工程建设。强力推进“城中村”拆迁改造，加强城市近郊建设的规划与管理。

　　(二)加快郑东新区建设步伐。力争引进项目累计达到180个，完成投资115亿元，建成区面积达到40平方公里，居住人口达到13万人以上。重点加快龙子湖、中心湖、南北运河、东西运河、起步区次干道、龙子湖外环路等基础设施建设。中央商务区内环30栋高层完工，外环10栋以上高层结顶。龙子湖高校区开工建设高校达到13所。加快龙湖南区、商住物流区、科技园区建设，部分项目建成投入使用。加快构建中央商务区金融聚集区和企业总部基地，着力培育发展现代物流、金融商务、总部经济、会展经济、高新技术、文化旅游等产业，抓紧完善商业、医疗、教育、市政环卫等配套功能。

　　(三)积极推进中小城市和小城镇建设。加大卫星城市规划和建设力度，不断完善城市功能，形成与中心城区规划上衔接、功能上对接、产业上联接的中小城市群，加快构建大郑州都市圈。充分尊重小城镇的历史文化特点、产业发展现状和自身区位优势，做好小城镇的总体规划和土地利用规划，加大资金、政策扶持力度，继续抓好重点镇建设。

　　(四)加强城市基础设施建设。扩大新建城区及城市周边区域的公交线路网覆盖面，使公交线路覆盖范围达到郑州市三环以外各郊区乡村。加快城区供水管网改造，提高供水质量。抓好郑东新区热电厂、兴隆铺热电厂二期扩建等供热工程建设，增强城市供热能力。积极拓宽天然气气源供应渠道，重点做好“川气入豫”项目协调和配套管网建设，努力缓解燃气供应紧张状况。加快马头岗污水处理厂建设步伐。加大老城区基础设施建设投入力度，改变部分老城区基础设施相对滞后的现状。

　　(五)进一步提高城市管理水平。坚持依法、科学、从严、有序管理城市，逐步改变传统的管理方法和模式，将数字城市建设和相关管理系统纳入城市管理工作中。深化城市管理体制综合改革，建立市与区合理分工、规范高效的城市管理框架，把城市管理的各项工作落到实处。巩固创建成果，加大环境卫生检查力度，稳定主次干道卫生保洁质量。做好城市垃圾的收集、清运和分类处理。加强户外广告设置管理，净化城市容貌。

　　四、进一步深化各项改革，着力发展开放型经济

　　不断加大改革力度，努力消除影响科学发展的体制障碍。强力推进国有企业改革，抓好中原制药厂等企业资产重组，加快电缆集团、金牛集团等重点企业改制步伐；完善法人治理结构，建立健全国有资本经营预算制度和经营业绩考核体系。把供销、民政、文化等系统所属企业作为改革重点，加快市直单位所属企业改制步伐。深化投融资体制改革。扩大企业投资自主权，规范政府投资行为，提高投资效益。继续加大融资力度，地产集团和建投、路桥公司要创新方式，扩大融资规模。引导企业积极引进战略投资资金，支持企业上市，力争年内有1-2家企业上市。深化完善部门预算、国集中收付、收支两条线、乡财县管等财政管理制度改革，规范政府采购行为。加大人事制度改革力度，稳步推进事业单位改革。加快市政公用事业改革，深入推进供销社体制创新和农村信用社改革。

　　继续实施开放带动战略，大力发展开放型经济。改进招商方式，更加注重招商的针对性，搞好专题性和专业性招商，拓宽招商引资渠道。筹备举办好黄帝故里拜祖大典，组织参加好第四届中国河南国际投洽会等重大招商活动。以郑东新区、开发区、出口加工区和各类工业园区为依托，发挥园区招商优势，不断扩大招商引资规模。抓好已签约重大项目的落实，强力推进加州工业城项目建设。加快优化出口商品结构，提高高技术、高附加值产品出口比重。以郑州出口加工区为依托，推动出口加工贸易持续增长。加大国际市场开拓力度，全方位拓展对外经济合作。大力发展对外工程承包和劳务合作，加快外派劳务基地建设。继续实施东引西进战略，加强骨干企业与东部企业的战略合作，积极承接东部产业转移；不断开拓中西部市场，鼓励优势企业到西部开发资源，建立生产基地。

　　五、坚持以人为本，全面推进社会主义和谐社会建设

　　积极协调各方面的利益关系，着力解决人民群众最关心、最直接、最现实的利益问题，更加注重社会公平和公正，促进社会和谐。

　　(一)千方百计扩大就业再就业，进一步完善社会保障体系。认真落实促进就业再就业的各项政策措施，进一步完善以市场为导向的就业新机制，努力开发多种就业岗位，重点解决下岗失业人员再就业问题和重组改制关闭破产企业的职工安置问题，有效抑制失业率，逐步实现社会就业比较充分的目标。加大对职业能力建设的投入，健全职业技能培训机制，力争年内培养各类技能人才12万人。加大劳动监察力度，切实维护劳动者合法权益。

　　努力建立制度完善、管理科学、体系完整、水平适度、运转高效的社会保障体系。继续扩大城镇社会保险覆盖面，加大社会保险费征缴力度。不断探索新的养老模式，完善城镇职工基本养老保险制度，确保企业离退休基本养老金按时足额发放。加强城镇基本医疗保险和失业、工伤、生育保险工作。切实解决推进城市化进程中失地农民的社会保障问题。加快农村敬老院建设，不断提高五保老人集中供养率。

　　(二)不断加大财政投入比重，大力发展各项社会事业。积极推进科技自主创新，加强技术创新体系建设，优化科技资源配置。加快产学研结合，促进科技成果转化。进一步改善办学条件，规划新建和改扩建一批中小学。继续支持普通高中建设，扩大招生规模，提高公办高中计划内招生比例。加快职业教育资源整合，启动郑州市职业教育园区一期工程建设，抓紧推进各县(市)职教中心建设，扩大中等职业教育规模。加强学前教育工作，提高幼教水平，启动郑州幼师升专工作程序。大力发展高等教育，加快郑州师专、中州大学合并升本步伐。

　　进一步完善公共卫生和医疗服务体系，加强公共卫生设施建设，提高应对突发公共卫生事件的能力。完成市二院整体搬迁，加快市疾病预防控制中心新建工程和市七院迁建等重点项目建设。全面完成县级动物防疫站建设任务，防止禽流感等动物重大疫病大面积发生。加快推进医疗卫生体制改革，大力整顿、规范药品价格和医疗服务秩序，认真解决群众看病难、看病贵问题。继续加强重大传染性疾病预防控制工作，加大对艾滋病知识的宣传和疫情监测力度。切实加强食品、药品安全监管，确保人民群众饮食用药安全。

　　加强人口和计划生育工作，加快建立利益导向机制，落实农村部分计划生育家庭奖励扶助政策，开展计划生育优质服务先进县、乡、村建设，切实加强对流动人口的计划生育管理服务工作，优化出生人口性别比结构，努力稳定低生育水平。抓好公共文化基础设施建设，做好市图书馆、考古研究院、郑州歌舞剧院等立项筹建工作，抓好基层文化设施建设，加快县级图书馆、文化馆达标升级。以创建“全国社区建设示范市”为目标，加大社区建设投入，完善社区功能，加强卫生、文化等服务体系建设，重点解决社区物业管理中存在的突出问题，构建和谐社区。大力开展群众性体育活动，加快竞技体育人才培养，提高竞技体育水平，办好郑州市首届农民运动会，加快建设郑州体育中学。

　　(三)加大环境保护力度，建设环境友好型社会。搞好环境综合整治，重点整治巩义、新密耐火材料群等重点区域，治理金水河、熊耳河、东风渠、贾鲁河、索须河、汜水河等河渠。关闭淘汰一批高能耗、重污染企业，关停单机1.2万千瓦及以下各类燃煤机组和单机2.5万千瓦纯凝气机组。积极推动13.5万千瓦以上火电机组建设脱硫装置。完成新密、登封、新郑污水处理项目，加快中牟、荥阳污水处理项目建设进度。加强环保地方立法工作，尽快制订《郑州市环境噪声污染防治办法》。进一步加强生态建设，强化森林资源保护和管理，科学规划建设农田林网，荒山、荒坡实行植被恢复，治理风沙源，推进嵩山山脉涵养林建设。加快森林生态城建设，完善沿黄生态林、自然保护区和湿地保护基础设施。

　　(四)切实加强安全生产和社会稳定工作，确保人民群众安居乐业。严格实行安全生产责任制，在全市重点行业、重点领域组织开展安全生产专项整治，加大对非法和不具备安全生产条件的小矿小厂、经营网点的关闭取缔力度。建立管理网络，落实监控措施，强化重大危险源监控和基层安全监管。组织开展经常性专项安全检查和督查，深入排查事故隐患。全面开展平安建设，进一步加强社会治安综合治理，深入开展“两严一创”活动，完善社会治安防控体系，建立健全预防和处置涉及公共安全突发事件的体制机制，持续开展命案和重大抢劫、盗窃案件的侦破，切实加强基层基础和队伍建设，营造城乡居民安居乐业的社会环境。落实关口前移、重心下移、标本兼治、多策并举等制度和措施，狠抓基础，注重法制，畅通信访渠道，加大督查督办力度，认真解决长期信访积案和重复信访问题，切实解决人民群众关注的热点难点问题，努力保持社会稳定。

　　(五)加强社会主义精神文明建设，激发全市人民干事创业、建设郑州的热情。大力弘扬以爱国主义为核心的民族精神和以改革创新为核心的时代精神，在全社会倡导基本道德规范。发挥工会、共青团、妇联的作用，深入开展群众性精神文明创建活动，争创文明单位、文明社区、文明村镇。进一步加强科普宣传教育，倡导科学精神和文明健康的生活方式。建设学习型社会，努力提高社会文明程度和公民整体素质。加强国防教育，积极支持部队和国防建设，深入开展“双拥”活动，增强军政军民团结，再创“全国双拥模范城”。继续做好民兵预备役工作，完善国防动员体系。

　　(六)坚持为民谋利，认真办好“十件实事”。通过广泛征集社会各界意见，确定了2006年为群众办好的“十件实事”：一是全市城镇12万人实现就业再就业，转移农村劳动力12万人。二是从新学年开始，免除城市低保家庭和全市农村学生九年义务教育阶段学杂费。三是改善农村居民生活条件，解决10万人的饮水安全问题，新建沼气池3万个、集雨节灌水窖1万个，贫困地区实现1万人脱贫。四是在全市农村全面推行新型合作医疗制度。五是新建、改造“村村通”道路600公里。六是从元月1日起，提高城乡低保标准。七是新开工经济适用住房60万平方米，竣工60万平方米，廉租住房覆盖率达到符合条件“双困家庭”100%。八是解决金水路交通堵塞问题。九是进一步改善城市生态环境，完成森林生态城工程造林10万亩，中心城区新增绿地200万平方米；净化市区大气环境，二级以上天数不低于280天。十是市区新建2所社会福利院。

　　继续做好统计、新闻出版、广播电视、外事、侨务、对台事务、民族宗教、社会科学、地方志、档案管理、气象和地震监测、人防等各项工作。

　　切实加强政府自身建设

　　适应新时期经济社会发展的需要，我们必须加快政府职能转变，进一步强化创新意识、服务意识、责任意识，弘扬不断进取、顽强拚搏、团结协作、勤政廉政精神，切实加强政府自身改革和建设，做到为人民服务、对人民负责、受人民监督、让人民满意。

　　(一)坚持改革创新，加快政府职能转变。继续推进行政管理体制改革，转变政府管理经济工作方式，进一步加快政企分开、政资分开、政事分开，注重履行政府社会管理和公共服务职能，把政府工作的着力点更多地放在发展社会事业和构建和谐社会上。继续深化行政审批制度改革，进一步清理、减少和规范行政审批项目，大力推行网上审批，建立严格的行政审批监督机制。优化组织结构，理顺部门分工，提高行政效能。

　　(二)坚持依法行政，建设法治政府。不断更新行政理念，完善行政程序，依法规范行政行为，逐步健全完善对行政权力的监督机制，自觉接受同级人民代表大会及其常委会的监督，接受人民政协的民主监督。大力推进政务公开，不断完善政务信息公开制度，增强政府工作的透明度，保障人民群众更多的知情权、参与权和监督权。进一步完善行政管理决策机制，健全集体领导和集体决策制度，健全对专业性、技术性较强的重大决策的专家论证、技术咨询、决策评估制度，对涉及群众利益的重大决策，都要进行公示和听证。建立决策失误责任追究制度和纠错改正机制，严格执行问责制，提高政府的公信力。加强行政监察和审计工作，全面推行领导干部经济责任审计制度。落实廉政建设责任制，深入开展反腐败斗争，做到廉洁行政。

　　(三)坚持执政为民，建设服务型政府。切实把实现好、维护好、发展好人民群众的根本利益作为政府工作的出发点和立足点，坚持以人为本，努力加快发展，让人民群众从发展中得到更多实惠。转变服务观念，改进服务方式，完善决策透明、信息公开、阳光操作的电政务服务体系，更好地为人民群众服务，更好地为基层、企业和社会公众服务。树立服务理念，变抽象服务为具体服务，认真解决土地征用、房屋拆迁、拖欠农民工工资等群众反映强烈的问题，认真纠正损害群众利益的不正之风。察民情，解民忧，多做群众受益的事情。

(四)坚持转变作风，建设高效政府。把求真务实的要求落实到政府工作的各个环节，一心一意谋发展，集中精力抓落实，做到雷厉风行。坚持讲实话、重实干、办实事、求实效，力戒形式主义，不做表面文章，克服官僚主义，防止急功近利。坚持改进会风、文风，严格控制和规范各种检查、评比、达标活动，减少不必要的应酬和事务性活动。厉行节约，反对浪费，建设节约型机关。坚持走群众路线，一切从实际出发，大兴调查研究之风，深入基层，深入群众，掌握全面情况，解决实际问题。认真贯彻实施《公务员法》，进一步完善公务员管理制度，切实加强公务员队伍建设。

　各位代表，我市经济社会发展已进入了一个新的历史时期，我们已站在新的发展起点上，任务繁重，时不我待，让我们携起手来，紧密团结在以胡锦涛同志为总书记的党中央周围，高举邓小平理论和“三个代表”重要思想伟大旗帜，解放思想，振奋精神，锐意进取，真抓实干，实现新跨越，再攀新高峰，为建设更加繁荣、和谐的大郑州而努力奋斗！
